# Identification of Characteristic Peptides of Casein in Cow Milk Based on MALDI-TOF MS for Direct Adulteration Detection of Goat Milk

**DOI:** 10.3390/foods12071519

**Published:** 2023-04-03

**Authors:** Yan Lu, Jinxia Dai, Sen Zhang, Junqin Qiao, Hongzhen Lian, Li Mao

**Affiliations:** 1 State Key Laboratory of Analytical Chemistry for Life Science, School of Chemistry & Chemical Engineering and Center of Materials Analysis, Nanjing University, Nanjing 210023, China; 2 Ministry of Education (MOE) Key Laboratory of Modern Toxicology, School of Public Health, Nanjing Medical University, Nanjing 211166, China

**Keywords:** goat milk authentication, allergen, bovine casein, specific peptide, matrix-assisted laser desorption ionization time-of-flight mass spectrometry

## Abstract

It is widely acknowledged that casein is an important allergenic protein in milk which may cause danger to customers. The identification and confirmation of caseins through mass spectrometry requires the selection of suitable characteristic peptides. In this study, by means of matrix-assisted laser desorption ionization time-of-flight mass spectrometry (MALDI-TOF MS), the three most representative specific peptides of caseins in cow milk were screened out with mass-to-charge ratios (*m*/*z*) of 830, 1195, and 1759, respectively. By comparing 2,5-dihydroxybenzoic acid (DHB) and *α*-cyano-4-hydroxycinnamic acid (CHCA) MALDI matrices, it was found that DHB was more suitable for peptide detection with the limits of detection (LODs) of 0.1 mg/L for *α*, *β*-casein. Furthermore, on the basis of verifying the characteristic peptides of casein from cow milk, this protocol was applied to goat milk authentication. Cow milk addition in goat milk was investigated by using the screened specific peptides. The results showed that the adulteration could be identified when the proportion of cow milk was 1% or more. When applied to inspect adulteration in five brands of commercial goat milk, specific peptides of bovine casein were detected in four of them. The method has the advantages of strong reliability, high throughput, simple preprocessing, and fast speed, which can provide powerful help for prewarning dairy allergen.

## 1. Introduction

Food allergies, especially dairy allergies, are threatening the health of allergic people. Most food allergies are type I hypersensitivity reactions, which are mediated by immunoglobulin E (IgE) [1]. Multiple allergen proteins can be present in the same allergenic food. Milk, for example, contains about 3.5% protein, usually divided into casein and whey. Of these proteins, eight were identified as allergenic, including *α*_s1_-casein, *α*_s2_-casein, *β*-casein and *κ*-casein from casein fractions, and *α*-lactalbumin, *β*-lactoglobulin, bovine serum albumin (BSA), and lactoferrin from whey fractions [2]. Caseins account for about 80% of milk protein [3]. Therefore, caseins could be used as a marker to determine whether there is milk in food, which can serve as a basis for people with allergies to judge food ingredients.

At present, there are many milk protein allergen detection methods, which can be divided into two categories in terms of detection objects. One category chooses DNA as the object, such as polymerase chain reaction (PCR) and loop-mediated isothermal amplification (LAMP) [4,5,6,7]. However, since these methods detect the non-allergenic DNA but not the allergen protein itself, it may be independent of the amount of protein in an unknown substrate. The other category focuses on allergen protein, which can mainly be further divided into immunoassay [8,9,10], chromatography [11,12,13,14,15], electrophoresis [16,17,18], and mass spectrometry. Immunological technology, for example, enzyme-linked immunosorbent assay (ELISA), is a highly specific, sensitive, rapid, and cheap method which identifies proteins or peptides by corresponding antibodies. However, it has the disadvantage of a high false positive or negative rate. In spite of many existing instrumental analytical methods for detecting allergens, problems such as laborious and time-consuming sample preprocessing, as well as high cost of sophisticated apparatus, cannot be ignored. Therefore, appropriate methods are needed for accurate and facile identification and confirmatory detection of allergens in dairy products.

The traditional LC-MS method [14] has the merits of high specificity and high sensitivity. It can clearly identify allergens or markers in various foods, but its defect lies in the long chromatographic separation time and tedious sample preparation to protect instruments from interference from sample matrices. Since the discovery of MALDI by Tanaka in 1988 and the construction of the first MALDI-TOF mass spectrometer in 1990, this technique has been widely used in various analyses. It is a soft ionization mass spectrometry technique, which is mainly used for macromolecular analysis. Due to its advantages of high sensitivity, high throughput, and rapid detection, MALDI-TOF MS is also being gradually applied in allergen proteins analysis [19]. In recent decades, this technology has been widely used to explore allergens and adulteration in the food industry, especially in dairy products [20,21,22,23,24,25,26]. Of all the applications based on MALDI-TOF MS, specific-peptide strategy is frequently adopted. In this method, unique and stable peptides of the allergen protein are selected and employed to detect whether the protein exists. The merit of this method lies in its high sensitivity, strong anti-interference ability, and simultaneous detection of multiple characteristic peptides without heavy workload or high analysis cost.

As is mentioned above, specific peptides of allergen proteins have a widespread application in food authentication, one of which is goat milk authentication. In terms of the market, due to the influence of production and other factors, the selling price of goat milk is higher than that of cow milk. Some merchants might mix the lower-value cow milk into the higher-value goat milk to obtain more profits. This adulteration will further increase the possibility of allergy. Therefore, a method to screen allergen proteins in milk and identify adulterated goat milk is in urgent demand. Casein, as the most abundant allergen protein in milk, can be used as a marker for the detection of goat milk adulteration. Sampelayo’s group [3] revealed that the total casein content in goat milk is comparable to that in cow milk, and several other caseins are even higher, as shown in Table S1. However, caseins in goat milk and cow milk are different in primary structure. The amino acid sequences of the two can be obtained by searching the Uniprot database—*α*_s1_-casein, for example (Figure S1). Additionally, this research group evaluated the allergenicity of goat milk versus cow milk, proving the hypoallergenicity of the former [27]. Russo et al. [20] explored a specific marker of bovine *β*-lactoglobulin based on MALDI-TOF MS, which might serve as a useful tool to detect adulteration in water buffalo ricotta. Calvano et al. [21] employed MALDI-TOF MS to examine goat milk and sheep milk adulteration. In their work, with necessary sample preparation, tryptic digestion, and MALDI analysis of plenty of raw milk and binary mixtures of cow milk, goat milk, and sheep milk, some specie-specific peptides were selected. Fifteen cow-specific peptides were screened out, two of which came from *β*-lactoglobulin, while others from caseins. A special MALDI matrix synthesized by them, *α*-cyano-4-chlorocinnamic acid (CCICA), was introduced to be better than the traditional CHCA matrix. However, this experiment was carried out via direct comparison of milk samples, screening out various peptides of different proteins with different sensitivities, thus probably introducing uncertainty and increasing the time and difficulty of analysis.

In view of the dominant number of peptides from casein selected in Calvano et al.’s work and the preponderant abundance of casein in milk, in the present work, to realize rapid and convenient inspection of adulteration in goat milk, characteristic peptides of caseins from cow milk were screened out, verified, and employed based on MALDI-TOF MS technology. Simple preparation of samples was performed to extract peptides in milk. Different common MALDI matrices were compared to choose the most suitable one to assist the ionization of specific peptides from caseins. Meanwhile, the reagent grade casein reference substance originating from bovine was analyzed and compared to make a certification. From all the peptides of bovine caseins, the most representative ones with high intensity and sensitivity were selected as specific peptides after confirmation. Eventually, the recommended representative specific peptides were applied in the examination of adulteration in goat milk. This method has the advantages of simple preparation, fast speed, high throughput, and accurate judgement.

## 2. Materials and Methods

### 2.1. Materials

*α*-Casein (P02662, P02663) from bovine, *β*-casein (P02666) from bovine, *α*-cyano-4-hydroxycinnamic acid (CHCA), and iodoacetamide (IAA) were purchased from Sigma-Aldrich (St. Missouri, MO, USA). 2,5-Dihydroxybenzoic acid (DHB) was purchased from TCI (Shanghai, China). Sequencing grade trypsin was acquired from Promega (Madison, WI, USA). Dithiothreitol (DTT) was from Roche (Mannheim, Germany). Acetonitrile (ACN), chromatographic grading, was obtained from Aladdin (Shanghai, China). Ammonium bicarbonate (NH_4_HCO_3_) was obtained from Macklin (Shanghai, China). Phosphatic acid (H_3_PO_4_, AR) was from SCR (Shanghai, China). Ultrapure water was obtained from Milli-Q Ultrapure Water Preparation System from Millipore (Burlington, MA, USA). The goat milk and cow milk were collected from Zhuomu Dairy (Hunan, China) and Mengniu Dairy (Inner Mongolia, China). Other goat milk samples were obtained at the local retailer. The cow milk used was skim cow milk if not otherwise specified.

### 2.2. Preparation of Standards

In all, 2 mg of casein was added to 2 mL NH_4_HCO_3_ solution (100 mmol/L) to prepare 1 mg/mL casein standards. For milk, a 30 μL sample was mixed with 970 μL NH_4_HCO_3_ (100 mmol/L) and then centrifuged at 16,873× *g*, 4 °C for 10 min, from which the supernatant was obtained. The concentrations of other reagents were as follows: DTT 500 mmol/L, IAA 500 mmol/L, trypsin 1 mg/mL. All solutions were prepared with 100 mmol/L NH_4_HCO_3_.

### 2.3. Alkylation and Enzymatic Digestion

In all, 0.5 mL of standard or supernatant, mixed with 20 μL of DTT solution, reacted at 75 °C in a water bath for 30 min. Then, the cooling mixture was added with 40 μL of IAA and reacted at room temperature in the dark for 30 min. The above solution was transferred to a 10 K ultrafiltration tube, centrifuged at 16,873× *g*, 4 °C for 20 min, and the solution at the bottom of the collection tube was discarded. Adding 100 μL of NH_4_HCO_3_ for washing, the mixture was centrifuged at 16,873× *g*, 4 °C for 20 min. The washing process was repeated three times. With the bottom collecting tube replaced, 100 μL of NH_4_HCO_3_ and 10 μL of trypsin (enzyme to protein mass ratio of about 1:50) were added to the ultrafiltration tube and incubated at 37 °C for 16 h. The peptide solution at the bottom of the collection tube was obtained by centrifugation at 16,873× *g*, 4 °C for 20 min.

In the test of LOD, the peptide solution (about 500 mg/L) was diluted with NH_4_HCO_3_ solution (100 mmol/L) to 2.5, 1, 0.1, 0.01 mg/L, respectively, and NH_4_HCO_3_ solution was used as a blank.

### 2.4. Mass Spectrometry

Two kinds of MALDI matrix (DHB and CHCA), with concentrations of 20 mg/mL and 10 mg/mL, respectively, were prepared with a mix of acetonitrile, phosphoric acid, and ultrapure water in the volume ratio of 50:1:49. Before analysis, a 1.0 μL sample was dropped on a MTP 384 target plate polished steel BC, and a 1.0 μL matrix after the sample dried.

To compensate for errors caused by the condition of instruments, casein reagents were analyzed simultaneously with samples each time for mass control. The mass error of samples in different batches was about 1 Da.

The identification experiment was carried out on an ultrafleXtreme MALDI-TOF/TOF mass spectrometer from Bruker Daltonics (Billerica, MA, USA). The laser emitted at 355 nm, and the acceleration voltage was 20 kV. Peptides were analyzed in reflection mode (positive ion). Triplicate to quadruplicate parallel determinations were carried out for each sample detected. Data on peptides were obtained by searching the database of PeptideMass, with Trypsin selected, one missed cleavage allowed, and a range of *m*/*z* 500–5000.

## 3. Results and Discussion

### 3.1. Selection of MALDI Matrices

The most vital part of MALDI is the use of a chemical matrix on the analyte in the form of small, laser-absorbing organic molecules. In the actual sample analysis, the selection of a suitable MALDI matrix is extremely important. Matrix selection should meet the following conditions: low sublimation temperature, non-reaction with the sample, co-crystallization with the sample, consistent absorption wavelength with laser, and good stability in vacuum [28].

DHB and CHCA are two commercial MALDI matrices commonly used in peptide analysis. According to Zhang et al. [29], in peptide mass fingerprint (PMF) analysis, CHCA is generally more sensitive at trace analyte concentrations, while DHB detects a greater number of peptides at higher analyte concentrations. However, the applicability of matrices is affected by different peptides. Therefore, a comparison of the two matrices was performed for all the tested samples including *α*-casein, *β*-casein, pure goat milk, pure cow milk, and goat milk mixed with cow milk at different volume ratios (1%, 5%, 10%, 50%). Taking *α*-casein, for instance, as is shown in Figure S2, in the same concentration, the sample assisted by the DHB matrix had the advantage in the number and intensity of peaks. When it came to the detection limit, peptides at the concentration of 0.1 mg/L could be detected with the assistance of DHB, while CHCA could not. As a result, DHB was chosen as a preferred matrix in this work for analyses of peptides. Another reason for choosing DHB was that it was an easily available MALDI matrix with a lower price compared to CHCA.

### 3.2. Screening of Specific Peptides

**Figure 1:**
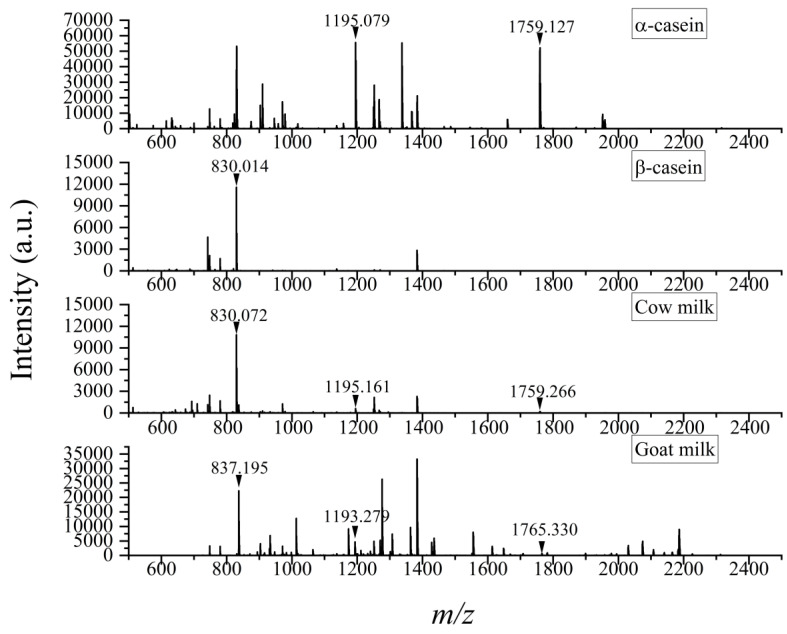
Peptide spectra of *α*-casein, *β*-casein, cow milk, and goat milk based on MALDI-TOF MS with DHB as matrix.

In recent decades, mass spectrometry (MS) has become the main technique for identifying proteins. The method used, PMF, is an important strategy of MS-based protein analysis, which identifies proteins by obtaining the profile of all peptides with different sequence lengths and then comparing it directly with theoretical PMF in the protein database [30]. In this present work, assisted by the DHB matrix, peptides of cow milk, goat milk, and casein from bovine were analyzed via MALDI-TOF MS. Based on the comparison of the peptide spectra (Figure 1), three peptides in cow milk with high intensity were selected as the tentative specific peptides of caseins from bovine. Two of them (*m*/*z* 1195, 1759) were found in the mass spectrum of *α*-casein, one (*m*/*z* 830) in *β*-casein, and none in goat milk. Through further searching the PeptideMass database, it was confirmed that these specific peptides were digestion products of caseins from cow milk, as was included in Calvano et al.’s work [21]. Identifying the most representative ones of the various peptides could reduce the time and difficulty of analysis, making the results more accurate. The database showed that the peaks at *m*/*z* 830, 1195, and 1759 were derived from *β*-casein, *α*_s2_-casein, and *α*_s1_-casein, respectively, with amino acid sequences of AVPYPQR, NAYPITPTLNR, and HQGLPQEVLNENLLR (Table 1). These peptides were recommended as the most characteristic peptides of bovine caseins.

Comparing the three characteristic peptides in cow milk, it was found that the intensity of the peptide sequencing AVPYPQR (*m*/*z* 830) from *β*-casein was stronger than that of the ones sequencing NAYPITPTLNR (*m*/*z* 1195) and HQGLPQEVLNENLLR (*m*/*z* 1759) from *α*_s2_-casein and *α*_s1_-casein owing to the relative higher abundance of its source protein in cow milk. In addition, their signal-to-noise (S/N) ratios, consistent with the intensity, were 1449, 95, 35, respectively.

As is known, the sites where trypsin cleaves protein are lysine and arginine, but it is not the case with proline present behind them because of steric hindrance. Considering that caseins in cow milk and goat milk differ from each other in amino sequence, the peptides are therefore supposed to vary. The screen-out of these bovine casein specific peptides provides convenience to identify the two kinds of milk. Moreover, for rapid detection methods such as ELISA mentioned above, the recommendation of the three most representative peptides also serves to provide antigen candidates.

### 3.3. Limit of Detection of Specific Peptides

**Figure 2:**
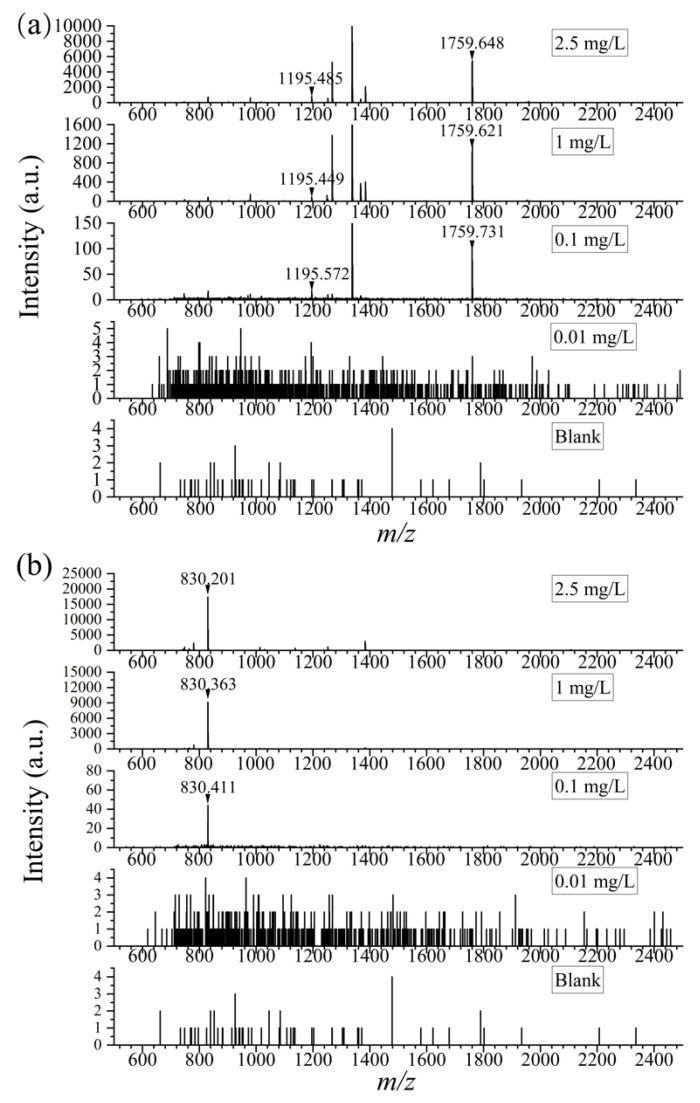
The mass spectra of specific peptides in *α*-casein (**a**) and *β*-casein (**b**) at different concentrations (0, 0.01 mg/L, 0.1 mg/L, 1 mg/L, 2.5 mg/L).

In food samples, the complex matrix and low abundance of proteins might cause difficulties in the detection of peptides. Therefore, a high sensitivity of the method is imperative. As is known, LOD is tightly connected with the sensitivity of a method. Commonly, methods with lower LODs are meant to have higher sensitivity. Thus, the problem lies in the test of LOD of the method. In the current work, the peptide solution digested from *α*-casein and *β*-casein was then diluted to 2.5, 1, 0.1, and 0.01 mg/L by 100 mmol/L NH_4_HCO_3_, respectively. Results showed that with the assistance of DHB, the selected specific peptides at *m*/*z* 1195 and 1759 could be detected in 0.1 mg/L *α*-casein trypsin digest, and the other one (*m*/*z* 830) in 0.1 mg/L *β*-casein digest. As shown in Figure 2, with the concentration of 0.1 mg/L, the S/N ratios of the three characteristic peptides were 8, 4, and 15, respectively, meeting the requirement of LOD which ought to be higher than 3.

### 3.4. Detection of Cow Milk Addition in Goat Milk

**Figure 3:**
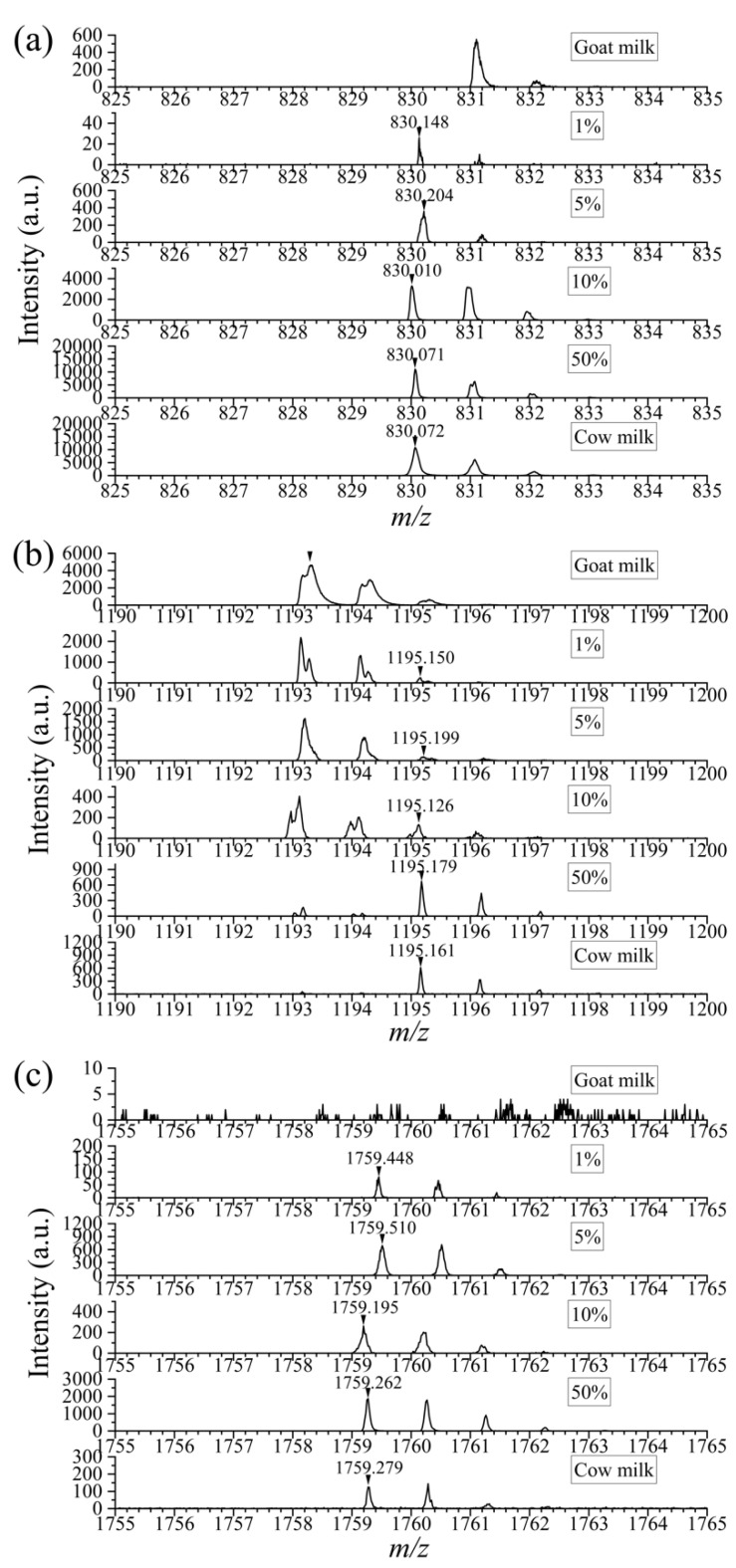
Comparison of mass spectra of specific peptides in goat milk mixed with cow milk at different volume ratios (0, 1%, 5%, 10%, 50%, 100%). *m*/*z* = 830 (**a**), 1195 (**b**), 1759 (**c**).

In view of the current phenomenon, there is an urgent need for proper methods to detect cow milk adulteration in goat milk to protect the health and interests of customers. The selected specific peptides played an important role. By mixing cow milk and goat milk in different volume ratios (1%, 5%, 10%, 50%), the specific peptides were analyzed via simple enzymic digestion and MALDI-TOF MS technology. Figure 3 shows that there was an interference peak of *m*/*z* 831 in pure goat milk, which was close to the specific peak of *m*/*z* 830 in cow milk. According to the result of database search, it was derived from *α*_s1_-casein in goat milk. However, due to the extra-high resolution of mass spectrometry (≥40,000), it could be seen that there was no peak of *m*/*z* 830 in pure goat milk initially. With the rise in spiking ratio, the peak intensity of *m*/*z* 830 accordingly increased. Based on the appearance of the peak (*m*/*z* 830), it could be judged that the addition exists. There was an isotope peak of *m*/*z* 1193 in goat milk itself at *m*/*z* 1195, the intensity of which was about 13.9% of the former according to the calculation based on data from six parallel experiments (Figure S3). Higher than that, the peak could be inferred as the specific peptide of bovine casein. For the peak of *m*/*z* 1759 and nearby peaks, they were not present in goat milk. In a word, based on the presence of these peaks, we can intuitively confirm the cow milk mixture. The results indicated that goat milk mixed with 1% cow milk could be detected with the DHB matrix. According to the general commercial consideration of adulteration, addition less than 5% has little profit. This sensitivity is therefore sufficient to cope with conventional adulteration of goat milk.

Considering that the cow milk used above was skim milk, further study was carried out using non-skim cow milk to check the effects of fat (Figure S4). The results showed that more than 1% adulteration could still be detected, which confirmed that the presence of fat had little interference on the detection of the specific peptides and verified the universality of this method.

### 3.5. Detection of Adulteration in Actual Samples

**Figure 4:**
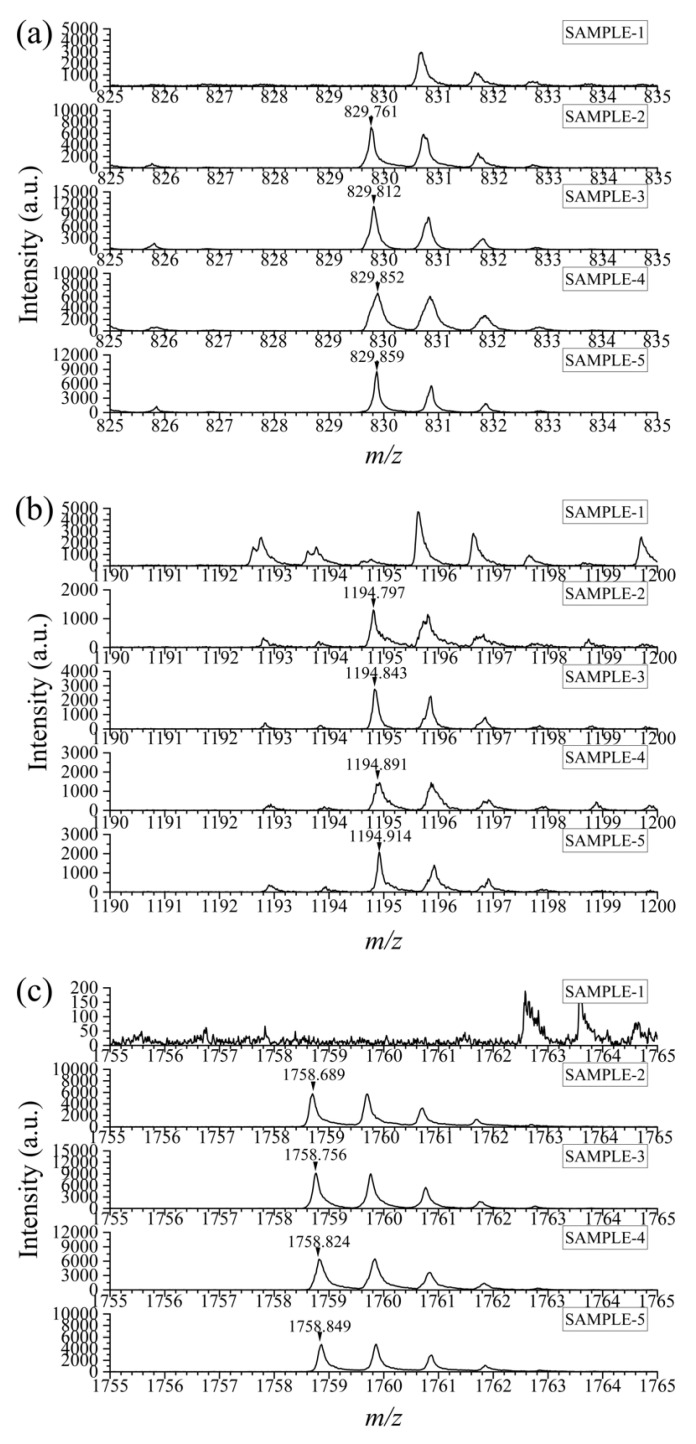
The mass spectra of selected specific peptides in goat milk samples from five brands. *m*/*z* = 830 (**a**), 1195 (**b**), 1759 (**c**).

Based on the selected specific peptides, goat milk samples from five brands in the market were analyzed. The preparation step of these samples was consistent with that of cow milk and goat milk mentioned above. The results showed that except for Sample 1, the specific peptides were detected in the rest of the samples (Figure 4). According to the ingredients list, in view of the presence of condensed milk for removing the odor of goat milk in Samples 2 and 4, which might derive from cow milk, it was hard to judge whether there was intentional adulteration in them even when more than 1% cow milk existed. However, the existence of bovine casein probably causes a certain risk of allergy to consumers. Meanwhile, for Samples 3 and 5, it could be inferred that more than 1% adulteration was present on evidence. The applicability of the proposed method is therefore proved.

## 4. Conclusions

To conclude, in this work, caseins in milk, which may cause allergies, were identified via MALDI-TOF-MS-based detection technology. Three peptides with *m*/*z* of 830 (AVPYPQR), 1195 (NAVPITPTLNR), and 1759 (HQGLPQEVLNENLLR) were screened out and verified by referring to bovine casein as specific peptides from cow milk. The LOD could reach 0.1 mg/L with the assistance of a common DHB matrix. When it came to the detection of cow milk adulteration in goat milk, 1% volume could be detected by the selected specific peptides. Due to the advantages of MALDI-TOF MS, including high sensitivity, high throughput, and rapid analysis, this method could serve as a useful tool for identification of caseins in cow milk and adulteration in goat milk, contributing to reducing the potential risk for people allergic to cow milk. This work also paves the way towards detecting adulteration in other high-value milks. Compared to previous work, the three most representative specific peptides from bovine caseins were recommended assisted by DHB, making goat milk authentication faster and more reliable. This might also serve as evidence for future application in rapid detection technology, such as ELISA kits or sensors.

## Figures and Tables

**Table 1:** The parameters of selected specific peptides.

Mass	Protein	Position	#MC	Peptide Sequence
830.4519	*β*-casein	192–198	0	AVPYPQR
1195.6793	*α*_s2_-casein	130–140	0	NAVPITPTLNR
1759.9449	*α*_s1_-casein	23–37	0	HQGLPQEVLNENLLR

Data from PeptideMass database. MC: missed cleavages.

## Data Availability

Data are contained within the article.

## Supplementary Materials

The following supporting information can be downloaded at: https://www.mdpi.com/article/10.3390/foods12071519/s1, Figure S1: Amino acid sequence of αs1-casein from cow milk (a) and goat milk (b); Figure S2: Comparison of peak quantity and intensity in the mass spectra of α-casein peptides in the same concentration with the assistance of two matrices (a) and comparison of the mass spectra of α-casein peptides at different concentrations assisted by DHB (b) and CHCA (c); Figure S3: The intensity ratio of isotope peak to the peak at *m*/*z* 1193; Figure S4: Comparison of mass spectra of specific peptides in goat milk mixed with non-skim cow milk at different volume ratios (0, 1%, 5%, 10%, 50%, 100%). *m*/*z* = 830 (a), 1195 (b), 1759 (c); Table S1: The protein content of goat milk and cow milk (S1).

## References

[1] Sampson H.A., O’Mahony L., Burks A.W., Plaut M., Lack G., Akdis C.A. (2018). Mechanisms of food allergy. J. Allergy Clin. Immunol..

[2] Petra L., Veronique P., Hans W. (2011). Development and validation of a method for the quantification of milk proteins in food products based on liquid chromatography with mass spectrometric detection. J. AOAC Int..

[3] Sanz Ceballos L., Ramos Morales E., de la Torre Adarve G., Díaz Castro J., Pérez Matínez L., Sanz Sampelayo M.R. (2009). Composition of goat and cow milk produced under similar conditions and analyzed by identical methodology. J. Food Compos. Anal..

[4] Wang Y., Yu Z., Liu Y. (2022). A high sensitivity method of closed-tube loop-mediated isothermal amplification developed for visual and rapid detection of cow milk adulteration. Int. Dairy J..

[5] Giglioti R., Okino C.H., Azevedo B.T., Gutmanis G., Katiki L.M., de Sena Oliveira M.C., Filho A.E.V. (2021). Novel lna probe-based assay for the A1 and A2 identification of β-casein gene in milk samples. Food Chem. Mol. Sci..

[6] Villa C., Costa J., Oliveira M.B.P.P., Mafra I. (2020). Cow’s milk allergens: Screening gene markers for the detection of milk ingredients in complex meat products. Food Control.

[7] Mayer H.K., Lenz K., Halbauer E.-M. (2021). “A2 milk” authentication using isoelectric focusing and different PCR techniques. Food Res. Int..

[8] Atrick W., Hans S., Angelika P. (2009). Determination of the bovine food allergen casein in white wines by quantitative indirect ELISA, SDS-PAGE, Western blot and immunostaining. J. Agric. Food Chem..

[9] Moen L.H., Sletten G.B., Miller I., Plassen C., Gutleb A.C., Egaas E. (2005). Rocket immunoelectrophoresis and ELlSA as complementary methods for the detection of casein in foods. Food Agr. Immunol..

[10] He S.F., Li X., Gao J.Y., Tong P., Chen H.B. (2018). Development of a H_2_O_2_-sensitive quantum dots-based fluorescent sandwich ELISA for sensitive detection of bovine *β*-lactoglobulin by monoclonal antibody. J. Sci. Food Agric..

[11] Pilolli R., Angelis E.D., Monaci L. (2017). Streamlining the analytical workflow for multiplex MS/MS allergen detection in processed foods. Food Chem..

[12] Cristina L., Elena A., Davide C., Giribaldi M., Lucia D., Cristiano G., Marco A., Carlo R., Cavallarin L., Gabriella G.M. (2016). Validation of a mass spectrometry-based method for milk traces detection in baked food. Food Chem..

[13] Mota M.V.T., Ferreira I.M.P.L.V.O., Oliveira M.B.P., Rocha C., Teixeira J.A., Torres D., Goncalves M.P. (2004). Enzymatic hydrolysis of whey protein concentrates: Peptide HPLC profiles. J. Liq. Chromatogr. Relat. Technol..

[14] Weber D., Raymond P., Ben-Rejeb S., Lau B. (2006). Development of a liquid chromatography-tandem mass spectrometry method using capillary liquid chromatography and nanoelectrospray ionization-quadrupole time-of-flight hybrid mass spectrometer for the detection of milk allergens. J. Agric. Food Chem..

[15] Monaci L., Losito I., Palmisano F., Visconti A. (2010). Identification of allergenic milk proteins markers in fined white wines by capillary liquid chromatography-electrospray ionization-tandem mass spectrometry. J. Chromatogr. A.

[16] Horká M., Šalplachta J., Karásek P., Roth M. (2022). Sensitive identification of milk protein allergens using on-line combination of transient isotachophoresis/micellar electrokinetic chromatography and capillary isoelectric focusing in fused silica capillary with roughened part. Food Chem..

[17] Mohamed H., Johansson M., Lundh Å., Nagy P., Kamal-Eldin A. (2020). Short communication: Caseins and α-lactalbumin content of camel milk (*Camelus dromedarius*) determined by capillary electrophoresis. J. Dairy Sci..

[18] Gasilova N., Gassner A.L., Girault H.H. (2012). Analysis of major milk whey proteins by immunoaffinity capillary electrophoresis coupled with MALDI-MS. Electrophoresis.

[19] Wieser A., Schneider L., Jung J., Schubert S. (2012). MALDI-TOF MS in microbiological diagnostics-identification of microorganisms and beyond (mini review). Appl. Microbiol. Biotechnol..

[20] Russo R., Rega C., Chambery A. (2016). Rapid detection of water buffalo ricotta adulteration or contamination by matrix-assisted laser desorption/ionisation time-of-flight mass spectrometry. Rapid Commun. Mass Spectrom..

[21] Calvano C.D., De Ceglie C., Monopoli A., Zambonin C.G. (2012). Detection of sheep and goat milk adulterations by direct MALDI-TOF MS analysis of milk tryptic digests. J. Mass Spectrom..

[22] Fanton C., Delogu G., Maccioni E. (1998). Matrix-assisted laser desorption/ionization mass spectrometry in the dairy industry 2. The protein fingerprint of ewe cheese and its application to detection of adulteration by bovine milk. Rapid Commun. Mass Spectrom..

[23] Cozzolino R., Passalacqua S., Salemi S., Malvagna P., Spina E., Garozzo D. (2001). Identification of adulteration in milk by matrix-assisted laser desorption/ionization time-of-flight mass spectrometry. J. Mass Spectrom..

[24] Nicolaou N., Xu Y., Goodacre R. (2001). MALDI-MS and multivariate analysis for the detection and quantification of different milk species. Anal. Bioanal. Chem..

[25] Di Girolamo F., Masotti A., Salvatori G., Scapaticci M., Muraca M., Putignani L. (2014). A sensitive and effective proteomic approach to identify she-donkey’s and goat’s milk adulterations by MALDI-TOF MS fingerprinting. Int. J. Mol. Sci..

[26] Sassi M., Arena S., Scaloni A. (2015). MALDI-TOF-MS platform for integrated proteomic and peptidomic profiling of milk samples allows rapid detection of food adulterations. J. Agric. Food Chem..

[27] Sanz Ceballos L., Sanz Sampelayo M.R., Gil Extremera F., Rodríguez Osorio M. (2009). Evaluation of the allergenicity of goat milk, cow milk, and their lactosera in a guinea pig model. J. Dairy Sci..

[28] Hillenkamp F., Peter-Katalinic J. (2007). MALDI MS: A Practical Guide to Instrumentation, Methods and Applications.

[29] Zhang C.J., Zhang H.X., Litchfield D.W., Yeung K.K.C. (2010). CHCA or DHB? Systematic comparison of the two most commonly used matrices for peptide mass fingerprint analysis with MALDI-MS. Spectroscopy.

[30] Thiede B., Hohenwarter W., Krah A., Mattow J., Schmid M., Schmidt F., Jungblut P.R. (2005). Peptide mass fingerprinting. Methods.

