# Supplementary material for: Identification of Characteristic Peptides of Casein in Cow Milk Based on MALDI-TOF MS for Direct Adulteration Detection of Goat Milk
Source: Foods. 2023 Apr 3;12(7):1519. doi: 10.3390/foods12071519 (PMC10094125; doi:10.3390/foods12071519)
Supplement: Supplementary file 1 [file foods-12-01519-s001.zip › foods-2260295-supplementary.pdf]

Supplementary Materials

Table S1. The protein content of goat milk and cow milk [S1].

Protein	Goat milk	Cow milk
	(g/100 g protein)	
casein	82.70	82.65
α_{s1}-casein	18.92	30.80
α_{s2}-casein	8.52	7.50
β+κ-casein	55.26	44.35

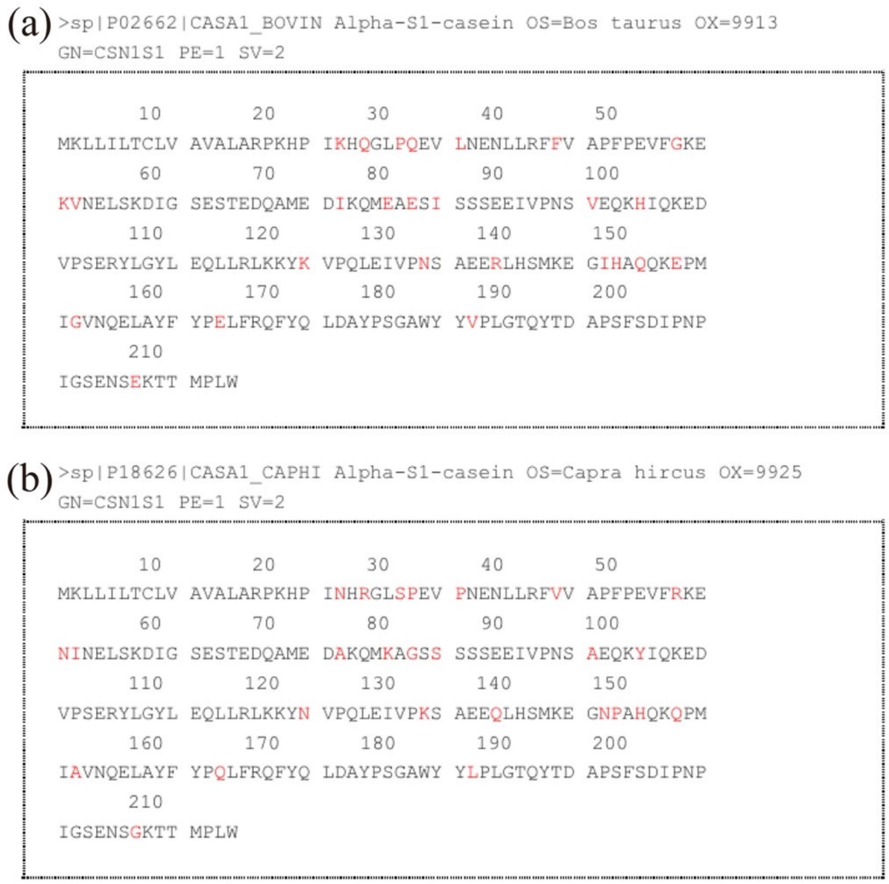

Figure S1. Amino acid sequence of α_{s1}-casein from cow milk (**a**) and goat milk (**b**).

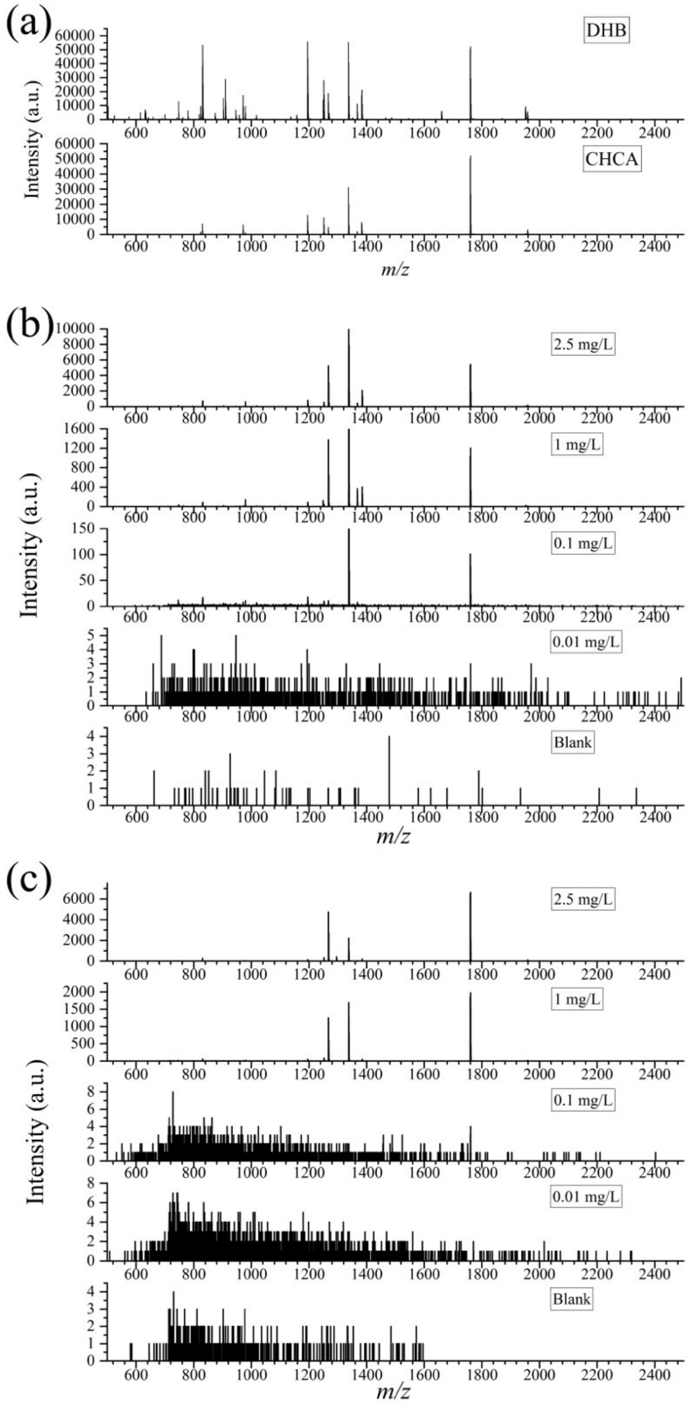

Figure S2. Comparison of peak quantity and intensity in the mass spectra of α-casein peptides in the same concentration with the assist of two matrices (**a**) and comparison of the mass spectra of α-casein peptides at different concentrations assisted by DHB (**b**) and CHCA (**c**).

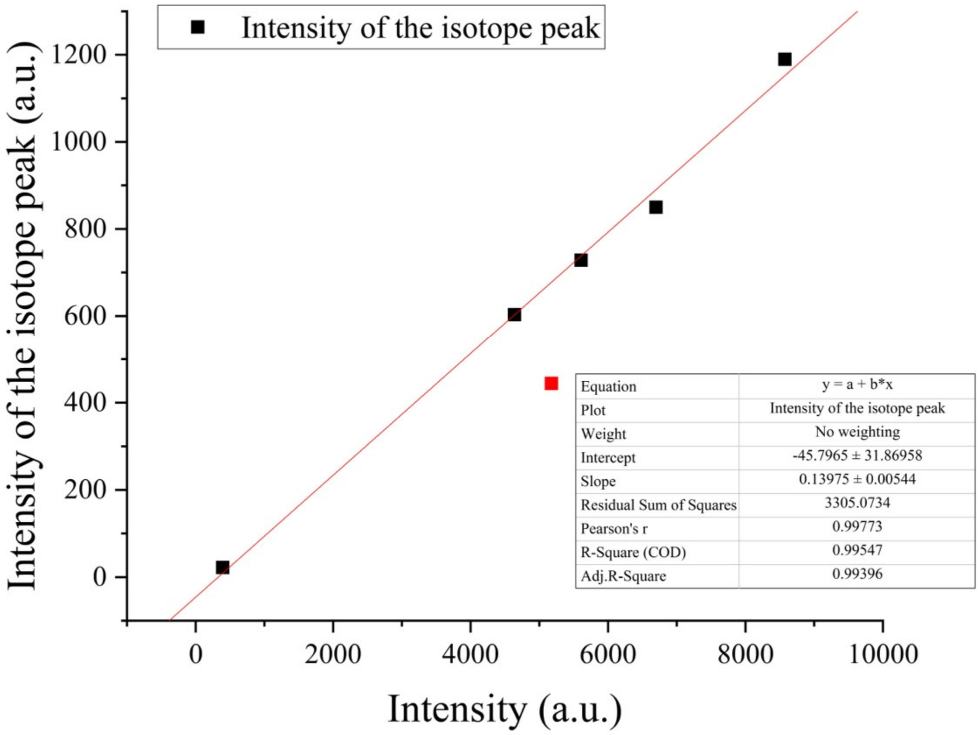

Figure S3. The intensity ratio of isotope peak to the peak at *m/z* 1193.

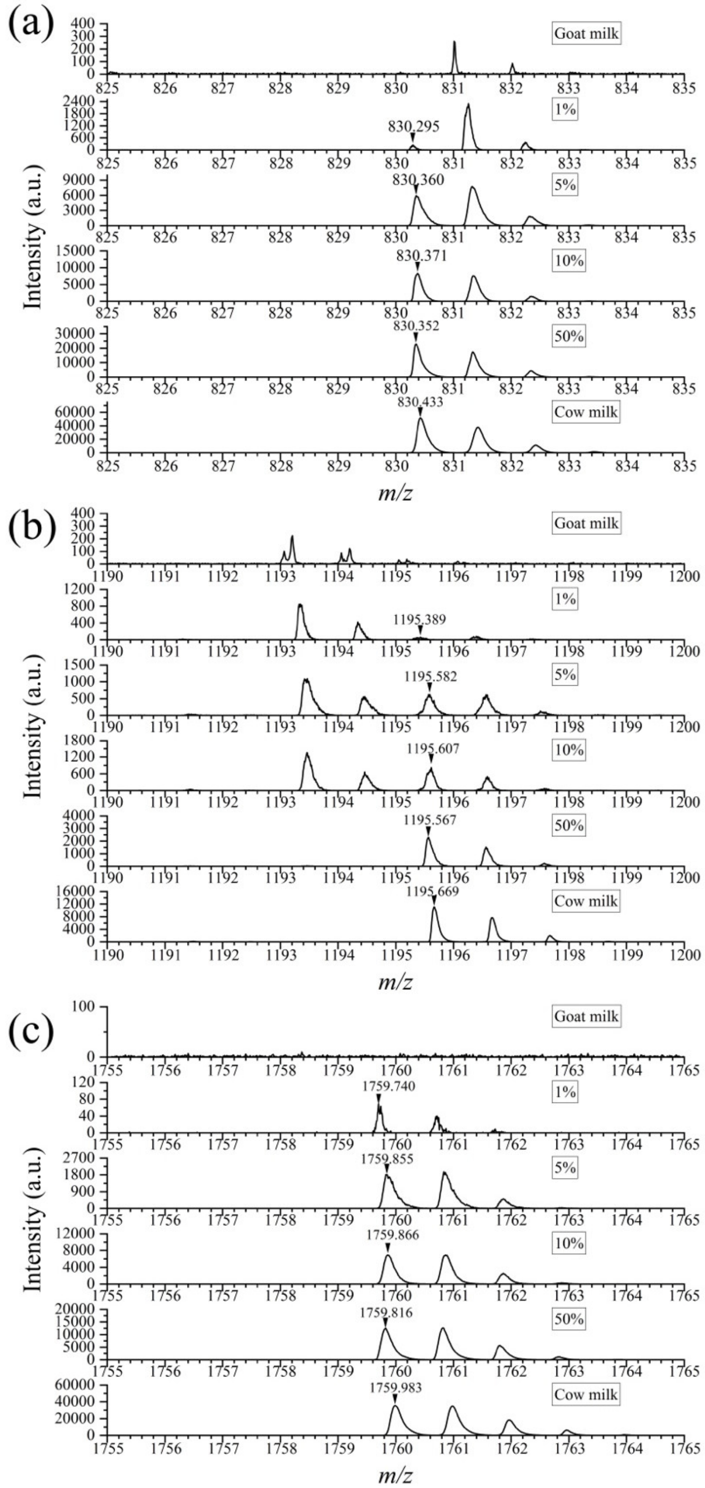

Figure S4. Comparison of mass spectra of specific peptides in goat milk mixed with non-skimmed cow milk at different volume ratios (0, 1%, 5%, 10%, 50%, 100%). *m/z* = 830 (**a**), 1195 (**b**), 1759 (**c**).

References

[S1] Sanz Ceballos, L.; Ramos Morales, E.; de la Torre Adarve, G.; Díaz Castro, J.; Pérez Matínez, L.; Sanz Sampelayo, M.R. Composition of goat and cow milk produced under similar conditions and analyzed by identical methodology, *J. Food Compos. Anal.* **2009**, *22*, 322-329. https://doi.org/10.1016/j.jfca.2008.10.020.
